# Leading with Impact: Transformational Leadership Among Women Surgeons – A Qualitative Study

**DOI:** 10.2147/JHL.S606964

**Published:** 2026-07-03

**Authors:** Laura Klösges, Barbara Maria Löfflad-Bürkin, Katarzyna Czabanowska

**Affiliations:** 1 Education Department, Swiss Tropical and Public Health Institute, Allschwil, Switzerland; 2 University of Basel, Basel, Switzerland; 3 Department of International Health, Care and Public Health Research Institute (CAPHRI), Maastricht University, Maastricht, the Netherlands; 4 Department of International Health, WHO Collaborating Center for Public Health Leadership and Workforce Development, Maastricht University, Maastricht, the Netherlands; 5 Department of Health Policy Management, Institute of Public Health, Faculty of Health Sciences, Jagiellonian University, Kraków, Poland

**Keywords:** transformational leadership, surgery, gender, qualitative research, competency framework

## Abstract

**Purpose:**

We explored the interdependence of contextual influences and transformational leadership by examining how women surgeons in leadership enact transformational leadership, navigate gender-related challenges, and influence surgical teams. The study further sought to propose key transformational leadership competencies among women surgeons.

**Patients and Methods:**

We conducted eight in-depth interviews with women surgeons in leadership positions. Data was analyzed by a sequential combination of thematic analysis and directed content analysis. An inductive approach and interpretation of themes at the semantic level guided thematic analysis. In a second step, transformational leadership served as the underlying theoretical framework for directed content analysis.

**Results:**

Participants acknowledge that “gendered perceptions of behavior”, the “surgical culture”, the “degree of congruence between inclusive policies and their implementation”, “endorsement of competence” and the need to “balance private and professional roles” influence how they reach, retain, and shape leadership roles. For women surgeons, transformational leadership competencies significantly shape their leadership style. They emphasize different competencies according to cultural, institutional, or individual context.

**Conclusion:**

The way women surgeons conceptualize and shape leadership practices corresponds to the four dimensions of transformational leadership. Organizational dynamics and cultural constructs are key influences on how women surgeons emphasize various competencies of transformational leadership behavior. We propose a transformational leadership competency profile based on these findings.

## Introduction

A surgeon leads when coordinating multidisciplinary teams in the operating room (OR) or emergency room (ER), during pre- and postoperative patient care, when mentoring students or residents, overseeing research or navigating difficult ethical decisions. Effective leadership is widely recognized as crucial to safe and high-quality surgical care.^1–3^ The “full range of leadership” theory by Bass and Avolio distinguishes transformational, transactional and passive leadership and is one of the most extensively studied leadership frameworks.^4^,^5^ Transformational leadership describes a leadership style in which leaders inspire and motivate their employees to move beyond personal interests to pursue a shared vision.^4^ Transformational leadership encompasses four dimensions: inspiration through role modelling and charisma (idealized influence), addressing the individual needs of each employee (individualized consideration), articulating a desirable common vision (inspirational motivation), and encouraging intellectual growth and creativity (intellectual stimulation).^6^

Transformational leadership is valuable across healthcare, but in surgery it is uniquely tied to OR team communication, resident development, and the shift away from traditional authoritarian surgical cultures.^7–9^ In the operating room setting, higher surgeon transformational leadership scores are associated with improved teamwork, specifically by increasing information sharing and voice behaviors, with the potential to improve efficiency and patient safety.^8^ In education, transformational leadership by supervising surgeons positively influences residents’ capability to handle the demands of surgery, access personal resources, and thus helps them “craft their jobs”.^9^

Despite an increasing number of women entering surgery, their representation in leadership positions remains disproportionally low.^10–12^ Systematic reviews have shown that women often encounter unfavorable workplace environments characterized by harassment, negative attitudes toward female professionals, and lower levels of support and respect^13^,^14^ Limited mentorship opportunities and rigid institutional structures not adapted for work–life flexibility further hinder their career progression.^15–18^ These challenges are often associated with a surgical culture that is characterized as hierarchical, male-dominated, with long working hours and an extensive work load.^14^,^19^,^20^ Both the increasing number of women entering surgery and a generational shift have led to a transitional change, but these characteristics still shape the surgical environment greatly.^21^ Collectively, these barriers contribute to higher attrition rates among women surgeons, and reduced diversity and equity in leadership positions. It potentially also influences patient outcomes, with studies reporting lower mortality rates among patients treated by female surgeons compared to male surgeons.^14^,^22^,^23^ Leadership directly influences whose voices impact resident training, clinical priorities, research agendas, and institutional culture. Therefore, a lack of diversity in leadership positions further reinforces structural barriers in surgery.

In surgery, several validated behavioral assessment tools focus on non-technical skills in surgery and incorporate leadership as a separate competence.^24–29^ A Delphi process produced the I-LEAD tool, an instrument defining the essential leadership behaviors for surgical residents leading inpatient teams, offering a shared mental model and assessment tool aligned with competency-based medical education (CBME).^30^ Literature shows that leadership curricula in surgery differ widely in content, format, and methods of outcome measurement and regularly lack evidence-based conceptual foundation.^31–33^ In a recent review, only one third of leadership development programs for surgical residents were rooted in standardized competency frameworks published by national surgical accreditation bodies.^34^ Traditional strategies for women surgeons leadership development have stressed individual adaptation, resilience, and personal skills, but evidence strongly suggests that leadership development extends beyond individual attributes to organizational constraints and institutional culture.^35^,^36^

There is limited understanding of how leadership should be conceptualized in surgery and whether specific leadership development frameworks are associated with behavioral or organizational outcomes.^37^,^38^ Research shows that women tend to lean more towards transformational leadership and in particular adopt more effective agentic and communal leadership behaviors compared to men.^39–42^ This is partly explained through role congruity theory, which argues that women’s gender roles align more closely with relational leadership styles.^43–45^ However, research on transformational leadership among healthcare professionals is limited, rarely differentiates by gender and often focuses on the nursing profession.^46–49^ Evidence also emphasizes outcome parameters such as job satisfaction, employee retention or patient safety rather than specific mechanisms.^50^ Cross-sector reviews show leadership development programs can strengthen women’s skills, confidence, and career advancement, but effects on gender equity remain insufficient without broader organizational change.^36^,^51^ Reviews of physician leadership programs do not explicitly report on the systematic integration of gender-specific content for women surgeons.^38^,^52^ Evidence remains underexplored on how women surgeons use transformational leadership to lead teams, overcome barriers, or which training approaches best support their leadership development.

Qualitative research in surgery is scarce, but particularly well-suited to examining the multifaceted interdependency between transformational leadership and contextual drivers. Exploring women surgeons’ perspectives allows for a nuanced understanding of the concept of transformational leadership in a women-specific and surgical context in greater depth than a quantitative approach. This research assesses the experiences of women surgeons in leadership to explore barriers to and competencies supporting a transformational leadership style within surgical contexts. It aims to conceptualize transformational leadership from a women surgeons’ perspective and develop a competency profile to inform leadership development initiatives in surgery. The research is guided by two questions: How does context affect women surgeons in realizing their transformational leadership potential? How can a transformational leadership training design incorporate contextual barriers and facilitators?

## Materials and Methods

The study employed a qualitative research design. Thematic analysis was selected to identify, analyze, and interpret recurring patterns in a study population for which only limited data exist to date.^53^ The data set for thematic analysis encompassed all instances across the entire data corpus that related to context or competencies for women surgeons in leadership roles. An inductive, data-driven approach and interpretation of themes at the semantic or explicit level guided this part of the analysis.^53^ The authors adopted a contextualist epistemological viewpoint, positioned between essentialist and constructionist perspectives.^53^ This approach recognizes participants’ accounts as reflecting their own experiences, but also acknowledges that social norms, such as gender norms, the surgical culture, and institutional contexts, shape personal experiences. In a second step, directed content analysis served to analyze and interpret the codes relating to competencies.^54^ Transformational leadership provided the underlying theoretical framework in a deductive category application.^4^,^54^ Bass’s four-dimensional model comprising idealized influence, individualized consideration, inspirational motivation, and intellectual stimulation guided the analysis.^55^ The study adopted a sequential combination of thematic analysis and directed content analysis to enrich data interpretation and limit researcher bias to non-conforming data or contextual aspects of the phenomenon of interest.^54^ We adhered to the Consolidated Criteria for Reporting Qualitative Research (COREQ) guidelines to guide the reporting of methodology and results (see appendix).^56^

**Figure 1:**
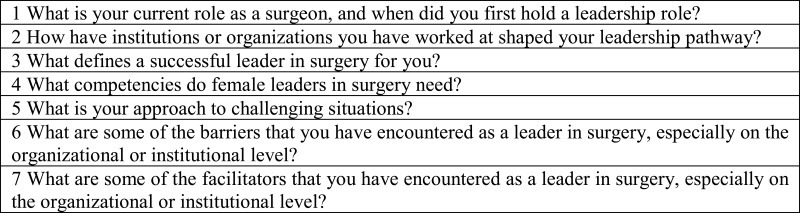
Interview questions (Transformational leadership among women surgeons, Switzerland, 2025).

Qualitative data were collected through in-depth interviews (Figure 1) with eight female surgeon leaders from May 2025 to September 2025. Participants were selected through purposive sampling to identify information-rich examples. Recruitment continued until thematic saturation was achieved, with no additional themes emerging from the data. The authors ensured that participants had diverse demographic backgrounds, including age, geographic location, and clinical specialty. After carefully considering the need to balance reporting transparency with protection of participant anonymity, we decided to limit the reporting of detailed participant characteristics (eg, country, specialty), given the small and potentially identifiable population of women surgeons in leadership in some countries of relevance. Participants practiced on five continents. Two participants worked in low-income and two in lower-middle-income countries, based on the World Bank classification.^57^ One participant worked in an upper-middle-income country, and three participants worked in high-income countries.^57^

Data collection was exploratory and participant-centered, allowing areas of focus to emerge organically during the interviews rather than being constrained by predefined categories. Semi-structured interviews were therefore not explicitly built around transformational leadership dimensions. An exploratory interview guide with open-ended questions was developed by LK and reviewed and revised by KC. Before data collection, the interview guide was piloted with two exemplary female surgeon leaders and adapted based on the feedback. Interviews lasted 45–60 minutes. Interviews were conducted by one member of the study team (LK) via Zoom in English and were audio- or video-recorded. All interviews were transcribed using NoScribe software.^58^ Transcripts were manually checked for verbatim transcription and anonymized by LK. All transcripts were read several times before beginning the coding process. Data management and coding were conducted by LK using MaxQDA software.^59^

A 6-step approach according to Braun and Clarke was adhered to during the initial thematic analysis of competencies: familiarizing with the data, generating initial codes, searching for themes, reviewing themes, defining and naming themes, and producing the report.^53^ Initial codes were drawn verbatim from participants’ accounts and organized into categories that reflected overarching themes. Next, directed content analysis was applied to the data. The codes identified from the data were organized into the categories “contextual drivers” or “competencies” of leadership for women surgeons. Context was defined as a tripartite framework of cultural, institutional, and individual factors. The initial codes generated by thematic analysis describing competencies were integrated with the four dimensions of transformational leadership: inspiration through role modelling and charisma (idealized influence), addressing the individual needs of each employee (individualized consideration), articulating a desirable common vision (inspirational motivation), and encouraging intellectual growth and creativity (intellectual stimulation).^6^,^55^ Codes were thus generated inductively from participant narratives and subsequently organized into the four domains of the transformational leadership framework to contextualize the findings. Themes were then developed from these categorized codes through directed content analysis informed by the transformational leadership framework. For example, one participant stated,

I try to build something to let them understand that I’m accessible as teacher, as mentor, and we have to work together to make things done. [P2]

This statement was coded as “accessibility” and “collaboration”, which contributed to the themes *individualized support of team members* and *adaptive leadership approach* within the category *individualized consideration*. Another participant described how

any complication or any mortalities, any morbidities that you get, should make you as a surgeon humble enough to know that you can make mistakes and learn from the mistakes. [P3]

This was coded as “being humble” and “learning from mistakes”. Both codes described nuances of the theme *authenticity*, while the focus on learning opportunities also related to *critical thinking* as an aspect of *intellectual stimulation*. This approach allowed for a systematic presentation of women surgeons’ leadership experiences within a well-established framework of leadership theory. The thematic map was iteratively reviewed and revised by the primary author, LK, and discussed with the senior author, CZ, to ensure internal consistency.

The study received a waiver from the Ethics Committee Northwest and Central Switzerland (reference: EKNZ 2025–00519). It declared that the project does not fall within the scope of the Human Research Act and therefore does not require its approval. Participation was entirely voluntary. Participants were informed of the study content, purpose and publication of anonymized responses beforehand and provided written informed consent before participation. Identifying information was removed from transcripts, and all data was securely stored.

## Results

**Figure 2:**
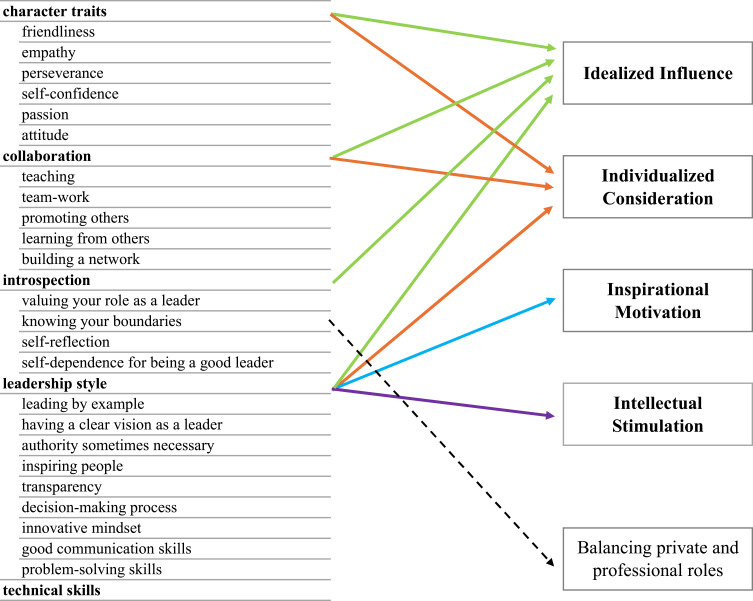
Initial thematic map derived from thematic analysis (left) and mapping of themes onto the four dimensions of transformational leadership using directed content analysis (right). Solid color-coded arrows show conceptual links between categories and dimensions; the dashed arrow indicates a code mapped to a theme describing context (Transformational leadership among women surgeons, Switzerland, 2025).

Initial interpretive thematic analysis revealed five themes encompassing leadership competencies across the data set: collaboration, technical skills, character traits, introspection, and leadership style. Apart from technical skills, which all participants viewed as a prerequisite of surgical leadership, these codes reflected all four dimensions of transformational leadership, particularly idealized influence and individualized consideration (Figure 2). The code “knowing your boundaries” better described contextual factors shaping women surgeons’ leadership. Figure 2 demonstrates how the initial thematic map of five themes and 24 codes maps onto the four dimensions of transformational leadership.

Directed content analysis of our initial thematic map revealed eleven themes describing competencies that participants regarded as essential for surgical leadership. They aligned with the four categories of transformational leadership (in italics). At the same time, participants emphasized that these leadership behaviors were strongly shaped by context. Five dominant themes emerged among three categories describing contextual influences shaping or being shaped by women surgeons’ leadership style (in italics). Participants reported how they apply specific competencies to meet these challenges and which competencies facilitate leadership for them. Based on these descriptions, we proposed a competency profile for women surgeons in leadership (Table 1).

**Table 1:** Competency Framework in Transformational Leadership Among Women Surgeons (Transformational Leadership Among Women Surgeons, Switzerland, 2025)

Competencies	Definition
Th1: Idealized Influence	
Building a network of trusted colleagues across the system	To build strong, reliable relationships within and outside the surgical ecosystem to serve as a resource of skills and knowledge and a support system for you and others
Professional commitment	To lead by example through passion, perseverance, and a consistent commitment to leadership
Authenticity	To consistently demonstrate an authentic leadership style and ensure balancing self-confidence and humility
Active listening and openness to feedback	To actively seek support, critique and feedback from others and integrate it into the decision-making-progress
Ownership of decisions and accountability	To demonstrate ownership of decisions, be transparent about mistakes and value accountability without blame
Th2: Individualized Consideration	
Adaptive leadership approach	To practice a consensus-building leadership style, but selectively adjust the approach along a collaborative-authoritarian continuum according to context
Individualized support of team members	To individualize support of team members, foster individual strengths and make everyone feel integral to success
Empathy in mentorship and patient care	To show empathy towards patients and team members and teach through a gradual escalation of autonomy
Th3: Inspirational Motivation	
Advocating for your values and vision	To advocate for the needs of patients and team members through openly communicating vision with authenticity, respect, and inspiration
Th4: Intellectual Stimulation	
Critical thinking	To critically re-assess decisions and actions and learn from them
Innovative mindset	To value creative problem-solving and explore innovative ideas
Intellectual curiosity	To consistently seek new perspectives and engage in a process of life-long learning

### Competencies in Transformational Leadership Among Women Surgeons (Table 2)

#### Idealized Influence

Participants report on how *building a network of trusted colleagues across the system* is crucial for navigating challenges and provides access to skills and knowledge. In addition, they describe how it transforms colleagues into “huge advocates, because they’ve developed that trust”, which in turn leads to greater “latitude to be authoritative” [P5]. They explore how they use their network to “start connecting” and to support others through opportunities such as “observerships for people, to get some grants and awards” [P6].

**Table 2:** Competencies in Transformational Leadership for Women Surgeons and Illustrative Quotes (Transformational Leadership Among Women Surgeons, Switzerland, 2025)

Themes and Competencies	Illustrative Quotes
Th1: Idealized Influence	
Building a network of trusted colleagues across the system	But if you think about building those relationships, they will change. And they will, over time, become huge advocates because they have developed that trust. And then (…) you have more latitude to be authoritative. [P5] But what you cannot get done, and this is something you cannot push, is your network. (…) You can be eager, you can be at all the congresses, but in the end, it’s repetitively showing what you can do, and repetitively being on time, being reliable. [P7]
Professional commitment	As a leader, you have to be exemplary. […] You try to set standards for yourself that are different from other people’s standards because you know that people are keeping a watch on you. [P1] I said to them, “Look at me, I am your teacher, your mentor, and I decided to leave my house to come to help you. Please, try to do the same. Try to come to learn with me.” [P2]
Authenticity	You really have to role model that every step of the way. That authenticity is really important, because people will know if you do not really care about them and about their opinion, and then it will not work. [P5] Part of your role as a leader is also definitely to recognize yourself, to know about yourself, to know your weaknesses and your strengths, to be able to deal with them in the context of being the leader. [P8]
Active listening and openness to feedback	I do not think it is important to agree with everything that people tell you. But the ability to listen, process it, and then make a decision on your own terms is good. [P4]
Ownership of decisions and accountability	There are far too many people who will take credit for the things that go well and step away if things are not going well. So, you have to own what you decide, because in the end, that’s what you are required to do as a leader. [P4] I think you need to be consistent. You know, you cannot say A and B. So, if you expect something from the people you are leading, you need to do the same. No exceptions. If you say “you have to be there for your patient”, then I have to be there for my patient. I think that if you do not do that, then people cannot trust you, they cannot believe you, you are not reliable. [P7]
Th2: Individualized Consideration	
Adaptive leadership approach	I usually believe in very consensus driven leadership, in which I will ask people (…) “How would you do this? And how would you do that?” [P4] You can have a more authoritative leadership style, for example in an emergency. I am a trauma surgeon, I am constantly seeing bad situations where I have to be the leader of the team. But I can still, in those moments, be authoritative, but still be open and welcoming input and be collaborative. [P5]
Individualized support of team members	Give space for every team member to be able to express themselves. Every team must have a role to play. Everybody must feel that they are part of a circle, that if they do not hold their end of the circle, then it’s no longer a circle. [P1] I try to build something to let them understand that I am accessible as teacher, as mentor, and we have to work together to make things done. [P2]
Empathy in mentorship and patient care	As you do it with passion, you want others to learn what you are doing and even do it better. […] I have not been selfish in imparting the same knowledge to others. [P3] A stepwise building of both skill and confidence, because one of the worst things you can do to a surgeon is to make them feel they do not have a safety net. (…) That stepwise escalation of both skills and confidence is essential. [P4]
Th3: Inspirational Motivation	
Advocating for your values and vision	Because the goal in my mind is a surgical department, or an institution even broader, is like rowing a boat together. If you want to go somewhere, you have to be coordinated to go to that end point. That really requires that direction. It requires authenticity from the leader that there is not going to be some ulterior motive, some other agenda. [P5] How to address people and communicate your goals and your way of going there. If you are not able to communicate properly, you will fail. [P8]
Th4: Intellectual Stimulation	
Critical thinking	I give myself space from the difficult situation to look at it from outside and see if I am able to bring in change. [P1]
Innovative mindset	Being in this situation, you have to innovate, you have to take a decision quickly, (.) you have to develop these leadership skills. If you do not have it, probably you will not be successful as a surgeon. [P2]
Intellectual curiosity	I think that it’s important for me, as a surgeon, as a person, to grow, to expand my mind, to keep searching. I am very curious. [P6]

To our interview participants, *professional commitment* means consistently demonstrating exemplary behavior, such as punctuality, respectful communication, and responsible use of authority. Passion for surgery, perseverance, and a “conscious commitment to leadership” [P4] are seen as indispensable. The women surgeons interviewed acknowledge their position as a role model, requiring integrity and high standards.

The data suggests that balancing self-confidence with self-doubts is an important aspect of *authenticity*. Participants mention humility towards one’s own power and limitations as a core competence. For our participants, this authenticity builds credibility and trust.

Participants offer several examples where *active listening and an openness to feedback* encourages others to speak up. They describe actively seeking and valuing diverse perspectives to “learn from them” [P7] prior to decision-making as essential.

Participants’ accounts point at the relevance of *ownership of decisions and accountability* for one’s own and the team’s outcomes: “you have to own what you decide to do and then see it through” [P4]. In their view, leaders must be transparent about successes and failures, constructively discuss errors and demonstrate consistent alignment between values, words, and action. One participant remarked that surgical practice trains these competencies from early on.

These five elements help women surgeons be viewed as respected leaders, inspire admiration, and encourage others to follow their example, all of which are central to the original dimension of idealized influence in Bass’s transformational leadership framework.

#### Individualized Consideration

Our data indicates that an *adaptive leadership approach* involves shifting between a collaborative and an authoritative leadership style in response to contextual influences, which participants describe as “two ends of the spectrum” [P5]. “Consensus-driven leadership” is the most common approach from participants [P4]. Open communication about shifts in leadership style seems to facilitate the challenging use of authority for women surgeons.

To our participants, an *individualized support of team members* means recognizing that some individuals need encouragement while others require boundaries. Participants foster individual strengths, making each team member “feel that they are part of a circle” [P1] and integral to success. In their view, transformational leaders inspire others by being accessible, empathetic, and genuinely committed to their team members’ personal and professional growth.

*Empathy in mentorship and patient care*, as reported in our data, emphasizes the challenges as well as the joy of sharing competencies and helping others to progress. Participants prioritize a gradual escalation of autonomy, skills, and confidence to ensure surgical residents are not forced to take “a leap of faith” and retain their “safety net” [P4].

These three aspects relate to how women surgeons engage with, support, and develop their teams individually, reflecting a key component of individualized consideration within transformational leadership theory.

#### Inspirational Motivation

Participants describe that *advocating for your values and vision* demands that leaders clearly articulate shared goals and values demonstrate that the chosen direction benefits everyone and aims at “making it better than it was yesterday” [P1]. One participant remarked that “it doesn’t necessarily have to be the same pathway as you, but maybe it can just inspire people to follow a pathway towards whatever they want.” [P6] Our data suggests that this is facilitated by clear communication with both team members and patients. The women surgeons interviewed advocate for the needs of their patients and team and convince those who may not initially be supportive with respect and inspiration.

Participants repeatedly emphasize the importance of inspiring and communicating a shared vision and outline how they enact this in practice. These findings align with the transformational leadership dimension of inspirational motivation.

#### Intellectual Stimulation

*Critical thinking* and an *innovative mindset* enable our participants to find creative solutions, reassess situations and learn from mistakes. They report challenging the status quo and actively promoting the same behavior in their team members. Participants state a dedication “to grow, to expand my mind, to keep searching” [P6] and a deep *intellectual curiosity* as main incentives to actively engage with colleagues from various backgrounds.

Participants describe critical thinking, an innovative mindset, and intellectual curiosity as key competencies, reflecting the intellectual stimulation dimension of Bernard M. Bass’s transformational leadership framework.

### Contextual Drivers of Transformational Leadership Among Women Surgeons (Table 3)

Women surgeons in leadership experience structural and cultural challenges deeply embedded in the surgical ecosystem. Barriers and facilitators influencing their leadership style and effectiveness can be viewed on an organizational, cultural, or individual level.

**Table 3:** Contextual Drivers in Transformational Leadership for Women Surgeons and Illustrative Quotes (Transformational Leadership Among Women Surgeons, Switzerland, 2025)

Themes	Illustrative Quotes
Th1: Organizational drivers and structural dynamics	
Degree of congruence between inclusive policies and their implementation	Very few institutions anywhere in the world would state those barriers. Usually very invisible. And when barriers are not dictated by an organization, (.) they are the ones that are coming from the people leading the organization. [P4] From an institutional point of view, no, theoretically on paper, you have the possibility to be in leading positions and be a woman and not have to work 100%. But the reality, how you then really implement things, is another issue. [P7]
Public and private endorsement of competence	That showed that they trusted me. I would call in a senior person only when I thought this operation will be difficult, “please come and check on me.” [P3] That’s probably the biggest thing, aside from backing you publicly, it’s directing those other relationships and signaling to those other relationships that they trust you. [P5]
Th2: Institutional culture and cultural paradigms	
Gendered perceptions of leadership behavior	We, the female, should acknowledge that there is some degree of challenge for us, and then our counterparts should probably be sensitive to know that. [P1] Even though I have all the required competencies, I will need to prove more. I will need to do more than a male with all my competencies. Because as a female, people always think “ah, you can’t do that. Ah, you are a female. Ah, this is not for a female.” (…) After having all the required competencies, after proving this, people expect you to do more. [P2]
Historical dimension of surgical culture	The concept of praise in public and shame in private: it’s very common in our morbidity and mortality culture and our historically hierarchical culture to blame in public. That destroys credibility for women much more rapidly and more severely than for men. [P5]
Th3: Resources and restraints on an individual level	
Balancing private and professional roles	We have so many roles, so you want to excel everywhere. (…) You will be an excellent leader, but at the expense of yourself. [P1] You also need your team in a personal way, that will support you for whatever you need; release of pressure, the nanny that can have the kid, or the person that can clean. You need help. [P6]

#### Organizational Drivers and Structural Dynamics

Advocacy and governance support for gender-diverse leadership presents a facilitator for participants, only if a *congruence between inclusive policies and their implementation* exists. This alignment was also framed by participants as “real leadership” [P6]. Participants embrace their responsibility as female role models and acknowledge the importance of such role models for their own careers.

Participants report that a consistent *public and private endorsement of competence*, also referred to as “entrustment with leadership” [P3] and “the authority to say what is best” [P1], facilitates leadership practice for them. Mentorship and sponsorship from both within and outside their institutions are valued “to help you think through challenges” [P5].

As a minor observation, both women surgeons from low-income countries noticed that a lack of resources, while not gender-specific, presents an additional burden.

#### Institutional Culture and Cultural Paradigms

Several of the women surgeons we interviewed view *gendered perceptions of leadership behavior* as a main barrier and report a double standard of evaluation: they perceive women leaders to start with lower perceived competence, having to work harder to prove themselves, and to lose credibility faster than their male counterparts. They recall a variety of situations where directness is perceived as aggressive or “abrasive” [P4], unlike their male counterparts, who are seen as confident.

Participants describe the *historical dimension of surgical culture* as an environment shaped by strong hierarchies and non-transparent accountability structures, long working-hours, an endorsement of agentic behaviors classically ascribed to men, and a “culture to blame in public” [P5]. Participants acknowledge both considerable progress and persistence of this culture. They report gender discrimination through exclusion from male-dominated networks or an “old boys’ club” [P4], but also overt gender discrimination in leadership circles.

#### Resources and Restraints on an Individual Level

Critical planning, having a supportive family, or an “army of home help” [P4] are highlighted as essential for *balancing private and professional roles*. Setting clear boundaries and prioritizing personal and family needs are identified as key individual facilitators by our study participants. Our data suggest that the expectation to excel in multiple roles often leads to personal sacrifice, but family support is seen as a great resource.

## Discussion

### Statement of Principal Findings

This study explored transformational leadership among women surgeons through the interplay of organizational and structural dynamics, cultural paradigms, and individual-level factors. The findings suggest that transformational leadership in surgery is not enacted as a static set of leadership traits, but rather as a highly context-dependent, relational, and adaptive process. Women surgeons described leadership as closely tied to authenticity, accountability, professionalism, collaboration, and interpersonal trust, while simultaneously navigating hierarchical surgical cultures, gendered expectations, and the high-pressure realities of patient care. Participants’ narratives reflected several dimensions of transformational leadership, particularly idealized influence and individualized consideration, while also highlighting contextual elements that extend beyond traditional transformational leadership frameworks.

### Strengths and Weaknesses of the Study

A major strength of this study lies in its qualitative and context-sensitive exploration of leadership among women surgeons, a group that remains underrepresented in leadership research despite persistent gender inequities in surgery.^10–12^ By integrating transformational leadership theory with the experiences of surgical practice, this study moves beyond decontextualized competency descriptions and situates leadership within the interaction of culture, organizational structures, and professional expectations. The findings provide nuanced insight into how leadership is enacted and perceived in high-risk clinical environments. At the same time, several limitations should be acknowledged. The qualitative design and sample characteristics may limit transferability across different healthcare systems, cultural settings, or surgical specialties. Furthermore, the study relies on self-reported experiences and perceptions, which may be influenced by retrospective interpretation or social desirability. Future studies incorporating observational approaches or perspectives from interdisciplinary team members may provide additional insight into leadership enactment in surgical practice.

### Strengths and Weaknesses in Relation to Other Studies, Discussing Particularly Any Differences in Results

The findings align with existing transformational leadership literature emphasizing relational leadership behaviors such as trust-building, communication, collaboration, and empathy.^8^,^39^,^48^,^60^ In particular, participants’ emphasis on authenticity and interpersonal relationships reflects core dimensions of idealized influence and individualized consideration. The findings also resonate with literature on non-technical skills in surgery, where communication, teamwork, and collaboration are increasingly recognized as essential for patient safety and team performance.^3^,^24^,^28^,^29^ However, compared to transformational leadership frameworks developed in public health or organizational settings,^48^,^61^ the surgical context appears to place greater emphasis on accountability, ownership of decisions, and the situational adaptation of leadership behavior. Participants repeatedly highlighted the necessity of “owning” decisions and managing their consequences in environments characterized by uncertainty, urgency, and potential harm to patients. This suggests that transformational leadership in surgery operates along a dynamic continuum between empowering and directive leadership behaviors depending on situational demands.

The study further highlights that transformational leadership in surgery cannot be fully understood independently of gendered leadership expectations. In line with role congruity theory,^44^,^45^ women surgeons described tensions between communal characteristics socially associated with women and the agentic leadership behaviors often expected within surgical environments. These tensions became particularly visible in high-risk situations requiring authority and rapid decision-making. Participants described strategies to navigate this perceptual incongruity, including explicitly communicating shifts in leadership style or balancing directive behavior with relational and communal approaches before or after critical situations. These findings suggest that women surgeons continuously negotiate professional legitimacy while simultaneously ensuring patient safety and effective team coordination. Networking also appeared to function differently for women surgeons compared to existing assumptions in leadership literature.^62^,^63^ Rather than primarily serving career advancement, networks were described as important sources of trust, legitimacy, and social support within environments where women leaders may encounter greater scrutiny.

The findings additionally indicate that existing transformational leadership models and surgical competency frameworks may not fully capture the realities of leadership in surgical settings.^25^,^29^,^30^,^64^ Competencies such as situational authority, authenticity, and relational trust were central to participants’ experiences yet remain underrepresented in many existing assessment tools and leadership taxonomies.^25^,^30^ This suggests that leadership in the operating room cannot be equated with leadership across all dimensions of surgical work and that leadership frameworks may require further contextual adaptation for high-risk healthcare environments.

### Meaning of the Study: Possible Mechanisms and Implications for Clinicians and Policymakers

The broader implications of this study extend beyond individual leadership competencies. While transformational leadership competencies may support women surgeons in navigating professional challenges, the findings suggest that leadership development alone is insufficient to address structural and cultural barriers within surgery. Participants emphasized the importance of inclusive organizational cultures, sponsorship, accountability mechanisms, and institutional support. In line with recent literature, the findings reinforce the notion that achieving greater equity in surgical leadership requires not “fixing women” by adapting them to existing systems, but rather transforming systems themselves through structural and cultural change.^35^,^36^ Leadership development initiatives should therefore not only focus on individual competency acquisition but also on organizational responsibility, inclusive cultures, and institutional accountability.

The competency profile identified in this study nevertheless provides a valuable foundation for integrating transformational leadership development into surgical education and leadership programs. The findings suggest that leadership training in surgery should incorporate relational competencies such as communication, trust-building, empathy, and collaboration, while also preparing surgeons to adapt leadership approaches situationally in high-pressure and high-risk contexts. Such approaches may help strengthen both leadership effectiveness and inclusive leadership cultures within surgery.

### Unanswered Questions and Future Research

Several unanswered questions remain and warrant further investigation. Future research could explore how transformational leadership competencies develop longitudinally across surgical careers and how leadership experiences differ across cultural or institutional contexts. Comparative studies involving male surgeons or interdisciplinary healthcare teams may further illuminate how leadership expectations are shaped by gender and professional culture.

Observational research examining leadership enactment directly within operating rooms and clinical environments could additionally deepen understanding of how transformational leadership behaviors are negotiated in practice. Finally, future studies should examine how organizational interventions, sponsorship structures, and institutional reforms may contribute to more inclusive and sustainable leadership environments within surgery.

## Conclusion

Women surgeons’ conceptualization of their leadership role reflects the four dimensions of transformational leadership: idealized influence, individualized consideration, inspirational motivation, and intellectual stimulation. Organizational and cultural dynamics shape how they emphasize specific competencies within this framework. Based on our findings, we propose a transformational leadership competency profile for women surgeons that highlights authenticity, accountability, professionalism, collaboration, and interpersonal trust. Strengthening the evidence on how women surgeons enact transformational leadership in practice is essential to advance leadership development and address persistent structural barriers in surgery.
